# Comparison of strain elastography, point shear wave elastography using acoustic radiation force impulse imaging and 2D-shear wave elastography for the differentiation of thyroid nodules

**DOI:** 10.1371/journal.pone.0204095

**Published:** 2018-09-17

**Authors:** Georgia Kyriakidou, Mireen Friedrich-Rust, Dimitra Bon, Ishani Sircar, Christopher Schrecker, Dimitra Bogdanou, Eva Herrmann, Joerg Bojunga

**Affiliations:** 1 Department of Internal Medicine 1, J.W. Goethe-University Hospital, Frankfurt, Germany; 2 Institute of Biostatistics and Mathematical Modeling, Faculty of Medicine, J.W. Goethe-University, Frankfurt, Germany; University of Montreal, CANADA

## Abstract

**Purpose:**

The aim of the study was to compare three different elastography methods, namely Strain Elastography (SE), Point Shear-Wave Elastography (pSWE) using Acoustic Radiation Force Impulse (ARFI)-Imaging and 2D-Shear Wave Elastography (2D-SWE), in the same study population for the differentiation of thyroid nodules.

**Materials and methods:**

All patients received a conventional ultrasound scan, SE and 2D-SWE, and all patients except for two received ARFI-Imaging. Cytology/histology of thyroid nodules was used as a reference method. SE measures the relative stiffness within the region of interest (ROI) using the surrounding tissue as reference tissue. ARFI mechanically excites the tissue at the ROI using acoustic pulses to generate localized tissue displacements. 2D-SWE measures tissue elasticity using the velocity of many shear waves as they propagate through the tissue.

**Results:**

84 nodules (73 benign and 11 malignant) in 62 patients were analyzed. Sensitivity, specificity and NPV of SE were 73%, 70% and 94%, respectively. Sensitivity, specificity and NPV of ARFI and 2D-SWE were 90%, 79%, 98% and 73%, 67%, 94% respectively, using a cut-off value of 1.98m/s for ARFI and 2.65m/s (21.07kPa) for 2D-SWE. The AUROC (Area under the Receiver Operating Characteristic) of SE, ARFI and 2D-SWE for the diagnosis of malignant thyroid nodules were 52%, 86% and 71%, respectively. A significant difference in AUROC was found between SE and ARFI (p = 0.008), while no significant difference was found between ARFI and SWE (86% vs. 71%, p = 0.31), or SWE and SE (71% vs. 52%, p = 0.26).

**Conclusion:**

pSWE using ARFI and 2D-SWE showed comparable results for the differentiation of thyroid nodules. ARFI was superior to elastography using SE.

## Introduction

Ultrasound is a reliable method for the detection of thyroid nodules, but not so accurate for the differentiation between benign and malignant thyroid nodules [1]. Fine needle aspiration biopsy (FNAB) is therefore used as an additional diagnostic method in the evaluation of thyroid nodules ≥ 10 mm in their greatest dimension, and could be recommended in the evaluation of nodules < 10 mm that have at least one feature of high suspicion in the ultrasound examination [2].

A hard or firm consistency upon palpation or ultrasound probe pressure is a classical criterion of malignancy [3]. In the past, this attribute was subjective and dependent on the examiner’s experience. However, reproducible qualitative assessment of tissue consistency became possible with the introduction of ultrasound-based elastography methods. In a meta-analysis of strain elastography (SE) a sensitivity of 92% and a specificity of 90% for the diagnosis of malignant thyroid nodules were reported [4]. Qualitative elastography was criticized for its operator dependency and as a result the evaluation of quantitative elastography was suggested [5].

Point Shear Wave Elastography (pSWE) using Acoustic Radiation Force Impulse (ARFI)-Imaging is an ultrasound-based elastography method enabling quantitative assessment of tissue stiffness. ARFI- Imaging was evaluated in previous studies as a non-invasive method for assessment of liver fibrosis and for evaluating the thyroid gland and thyroid nodules [6][7].

2D-Shear Wave Elastography (2D-SWE) is a new promising and useful 2D-elastography technique for the evaluation of thyroid nodules. In a previous study the combination of B-Mode ultrasound and 2D-SWE achieved the highest specificity (97%) with a sensitivity of 81.5% for the differentiation between benign and malignant thyroid nodules [8].

The aim of this study is to compare the three ultrasound-based elastography methods SE, ARFI and 2D-SWE for the differentiation between benign and malignant thyroid nodules using cytological/histological assessment as a reference method. SE is a qualitative elastography method, while ARFI-Imaging and 2D-SWE are quantitative elastography methods. These three methods might therefore supplement each other in the evaluation of thyroid nodules.

## Materials and methods

### Patients

The study was performed in accordance with the Declaration of Helsinki and approved by the local ethical committee of the Goethe University Frankfurt. All patients provided informed written consent to participate in the study. The study is a prospective study and was performed from May 2014 to October 2015. Patients were recruited from the Endocrinology department of the Goethe University Hospital in Frankfurt. All patients with thyroid nodules referred to our endocrinology department were included in the study, providing thyroid nodules were detected, which were due to undergo either FNAB or surgery within the study period. The included patients formed a consecutive series. Some patients refused FNAB and received thyroid surgery as a first-line treatment. Inclusion criteria were the presence of a thyroid nodule ≥10 mm, and either FNAB of this nodule performed within the preceding 6 months, or performed earlier on condition that nodule size did not change significantly between ultrasound examinations (modification <50% in volume or<20% in diameter), or FNAB and/or surgery planned at the time of ultrasound examination and performed within the study period. Exclusion criteria were completely cystic lesions, indeterminate cytology by FNAB without repeat FNAB, and suspicious or malignant cytology by FNAB without thyroid operation within the study period.

All patients received an ultrasound scan of the thyroid gland followed by SE and 2D-SWE, and all patients except for two received ARFI-Imaging. Cytology or histology was used as a reference method for the diagnosis of benign or malignant thyroid nodules. Ultrasonography and elastography were performed by 3 clinicians experienced in the use of strain elastography and point shear wave elastography using acoustic radiation force impulse imaging through their daily clinical practice in the endocrinology department of Goethe University Hospital in Frankfurt, and received additional training in 2D-shear wave elastography by performing 50 elastography ultrasound scans before the initiation of the study. All clinicians had at least 2 years of experience in B-mode ultrasound and at least 1 year of experience in elastography. Each patient was examined by one physician. Each patient was examined with the three elastography methods on the same day. The examination order was not predefined and depended on the availability of the three machines on each day.

### Fine needle aspiration biopsy (FNAB)/Histology

All patients received either FNAB and/or thyroid surgery for the diagnosis of benign nodules, and thyroid surgery only for the diagnosis of malignant nodules. FNAB was performed with a 25-gauge needle attached to a 10ml-syringe. Patients with suspicious or malignant cytology were referred for surgery. Some patients refused FNAB and received thyroid surgery as a first-line treatment. Patients with indeterminate cytology without repeat FNAB, and patients with suspicious or malignant cytology by FNAB without thyroid operation within the study period, were excluded from the study.

Cytology and histology were examined by 3 experienced pathologists with at least 5 years of working experience.

### Conventional ultrasound (B-mode and Doppler)

All patients received a B-mode ultrasound examination of the thyroid gland, and most patients received color Doppler ultrasound using a 9-MHz transducer (Hitachi-EUB 900, Hitachi, Tokyo, Japan and Siemens S2000 Erlangen, Germany). Color Doppler ultrasound enables the determination of intranodular vascularisation. Color Doppler ultrasound patterns were defined as absence of blood flow (pattern 0), minimal internal flow without a peripheral ring (pattern 1), peripheral ring or flow but minimal or no internal flow (pattern 2), peripheral ring or flow and a small to moderate amount of internal flow (pattern 3), extensive internal flow with or without a peripheral ring (pattern 4). [9] Experienced examiners blinded to the results of cytology performed the ultrasound scans. Thyroid nodules were evaluated for size, volume, echogenicity, echotexture, the presence/absence of a halo sign, and the presence/absence of microcalcification and/or macrocalcification.

### Strain elastography (SE)

**Fig 1:**
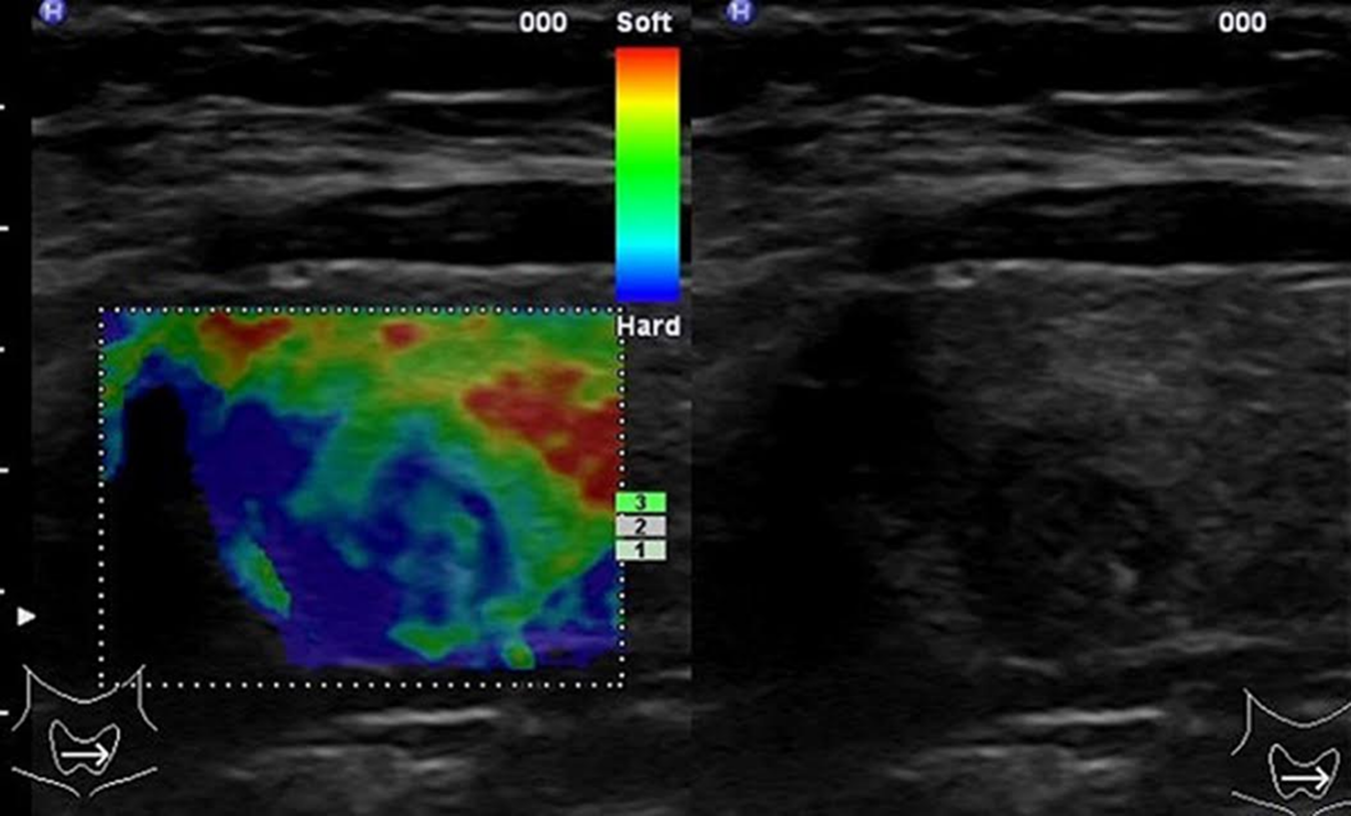
SE of a thyroid nodule in the right thyroid lobe. Histology revealed papillary carcinoma.

Strain elastography (Hitachi Strain Elastography [HI-SE], Hitachi Medical Corporation, Japan) is an ultrasound-based elastography method enabling qualitative assessment of tissue stiffness with conventional ultrasound probes. Calculation of the tissue elasticity distribution is based on the strain (which is the tissue deformation produced by external palpation) and stress of the examined tissue[10]. Strain detects the local deformation under slight pressure and displays it as a relative value in comparison to the strain values of the different tissues within the region of interest. The examination results are represented as color-coded images over the conventional B-mode image (blue = hard, red & green = soft tissue) (Fig 1) [11]. A previous study revealed that predominantly or completely blue nodules were suggestive of malignancy, while predominantly or completely green nodules were more likely to represent benign nodules [12]. SE was performed with the EUB-900 ultrasound-system (Hitachi, Tokyo, Japan) using the 9-MHz probe. The probe was placed on the neck and light pressure towards the neck was applied. A pressure of 3–4 units on a scale of 0–6 arbitrary units was applied for the measurement. The operator selected the region-of-interest (ROI) for the elastography examination including the nodule and surrounding normal thyroid tissue. SE measures the relative stiffness within the ROI using the surrounding tissue as reference tissue. The classification of elasticity was as follows: elasticity score (ES) 1: the nodule is displayed completely in green (soft); ES 2: the nodule is displayed predominantly in green with few blue areas; ES 3: the nodule is displayed predominantly in blue with few green areas; ES 4: the nodule is displayed completely in blue (hard). The strain ratio was calculated for each nodule by dividing the strain value in the surrounding healthy thyroid tissue by the strain value in the thyroid nodule, in order to provide a semi-quantitative analysis. The strain ratio between a nodule and the surrounding healthy thyroid tissue has also been proposed to differentiate benign from malignant nodules [10].

### Point shear wave elastography using acoustic radiation force impulse (ARFI)-imaging

**Fig 2:**
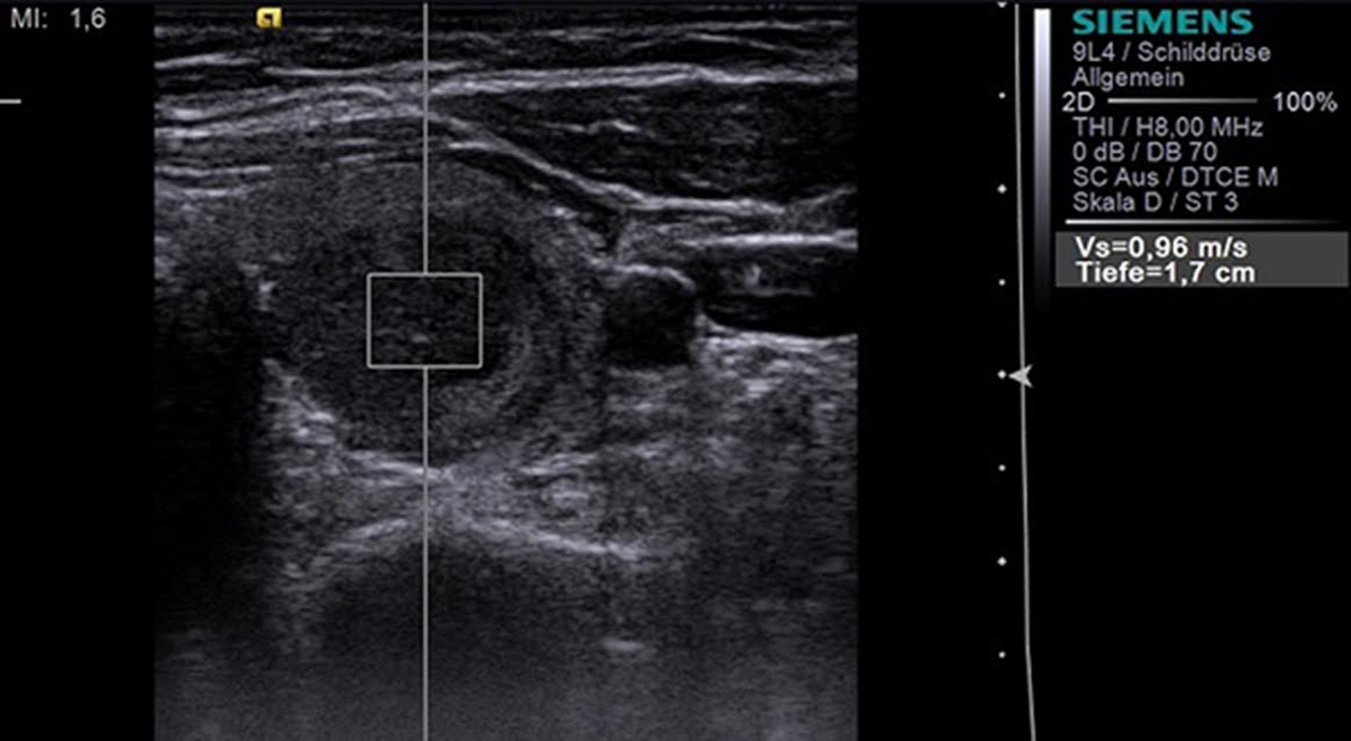
Acoustic Radiation Force Impulse Imaging (S2000, 9L4 probe at 9MHz) with the ROI placed within a hypoechoic thyroid nodule in the left thyroid lobe measuring a velocity of 0.96 m/s. Histology revealed benign nodule.

ARFI-Imaging (Virtual Touch™ tissue quantification, Siemens ACUSON S2000) is an ultrasound technique which involves targeting of an anatomic region to be interrogated for elastic properties with a Region-of-Interest (ROI) cursor, while performing real-time B-mode imaging. The acoustic pulses generate localized tissue displacements and as a result the tissue at the ROI is mechanically excited. The displacements cause lateral shear wave propagation which is tracked using laterally positioned ultrasound tracking beams [13]. The shear wave velocity of the tissue can be reconstructed by estimating the maximum displacement at each lateral location [14]. The shear wave propagation velocity is proportional to the square root of tissue elasticity [15]. Results are expressed in m/s. ARFI-Imaging was performed with a 9L4 linear ultrasound probe at 9-MHz for B-mode imaging. Five successful measurements were performed with the ROI placed within the thyroid nodule (Fig 2), and five successful measurements were performed with the ROI placed in the healthy thyroid gland away from thyroid nodules. The ROIs were placed inside the nodule, including as much nodule area as possible in all frames of the cycle. The ROI size was fixed (5 x 5 mm) and could not be adjusted. The strain ratio was calculated for each nodule by dividing the strain value in the surrounding healthy thyroid tissue by the strain value in the thyroid nodule.

### 2D-Shear wave elastography (2D-SWE)

**Fig 3:**
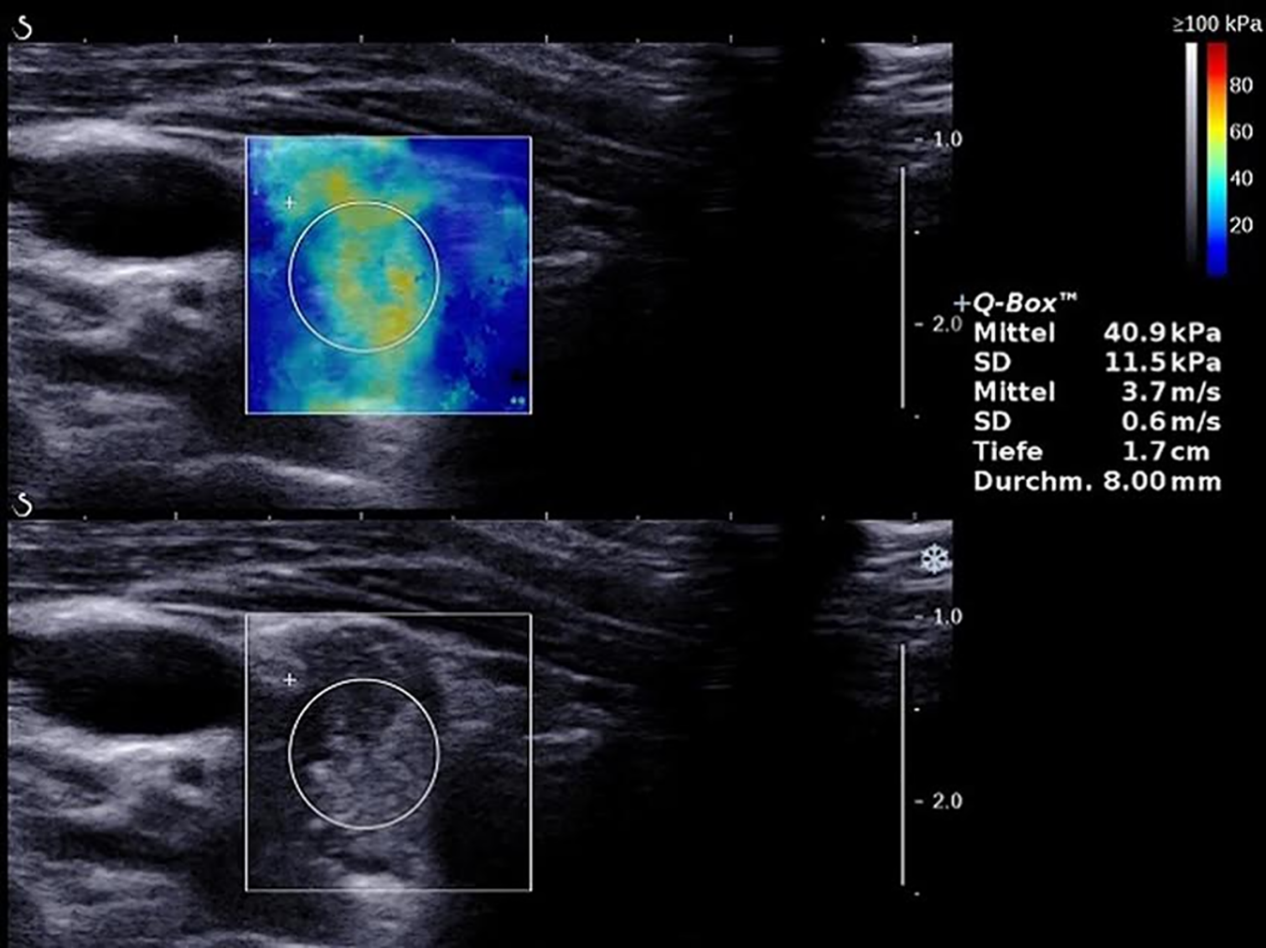
2D-SWE with the ROI placed within a thyroid nodule in the right thyroid lobe measuring a velocity of 3.7 m/s. Histology revealed papillary carcinoma.

2D-Shear Wave elastography (2D-SWE) is a most recently developed 2D-elastography technique which uses acoustic radiation force created by focused ultrasonic beams to induce mechanical vibrations. The induced tissue displacement creates shear waves which spread through the tissue of interest. Propagation of the resulting shear waves, captured by high frequency ultrasound imaging sequences, enables the assessment of tissue elasticity. Shear wave velocity is used for the assessment of tissue elasticity [16]. Elasticity values are displayed in a color-coded image, which presents softer tissue in blue and harder tissue in red. The color scale is quantitative with values expressed in kPa ranging between 0–100 kPa. The data are recorded as elasticity using kPa and as shear wave speed in m/s. 2D-SWE was performed using the Aixplorer ultrasound (Supersonic Imagine, Aix-en-Provence, France). At least five measurements were performed for each nodule and for the surrounding thyroid tissue. The median value of the 5 measurements was calculated. The mean value and the standard deviation were recorded. One region of interest (ROI) was placed over the thyroid nodule (Fig 3) and one ROI within the healthy thyroid tissue. The ROIs were placed inside the nodule, including as much nodule area as possible in all frames of the cycle. The ROI could be adjusted in size and position depending on the size of the nodule. The strain ratio was calculated for each nodule by dividing the strain value in the surrounding healthy thyroid tissue by the strain value in the thyroid nodule.

### Statistical analysis

Statistical analysis was performed using BiAS for Windows (version-10.12, epsilon 2014, Frankfurt, Germany). For ARFI-Imaging and 2D-SWE the median of all 5 measurements per nodule or healthy thyroid gland was calculated and used for further analysis. Clinical and laboratory characteristics of patients were expressed as mean ± SD, median and range. Comparison of patient characteristics was performed using the parametric t-test and Mann–Whitney U test for quantitative values, and the chi-squared test, Craddock-Flood test or Fisher’s exact test for qualitative characteristics. The logarithmic values of the parameters were normally distributed for both groups of malignant and benign thyroid nodules, thus non-parametric comparisons were not necessary. P-values < 0.05 were considered statistically significant. Sensitivity and specificity of the three elastography methods were compared using the chi-squared test.

The diagnostic performance of SE, ARFI-Imaging and 2D-SWE was also assessed by receiver operating characteristic (ROC) curves. The ROC curve represents sensitivity versus 1-specificity for all possible cut-off values for prediction of the different fibrosis stages, respectively. Cut-off values for SE, ARFI and 2D-SWE for the diagnosis of malignant thyroid nodules were defined using Youden'sindex [17], and sensitivity, specificity, positive and negative predictive values, and positive likelihood ratio were calculated from the same data.

## Results

Seventy patients with a total of 95 thyroid nodules were examined and met the inclusion criteria. Nine thyroid nodules were excluded because of non-diagnostic aspirate on FNAB without repeat FNAB or surgery during the study period. Two additional patients were excluded because of suspicious aspirate on FNAB without surgery during the study period. Therefore, 62 patients (21 men and 41 women) with a total of 84 examined nodules remained in the final analysis. Patient characteristics are shown in Table 1.

**Table 1:** Patient characteristics.

Characteristics	All 62 Patients with 84 nodules	52 Patients with 73 benign nodules	10 Patients with 11 malignant nodules	P-value
Patient age (years)				0.037
Mean +/- SD (range)	50±14.9 (22–82)	51.8±14 (30–82)	41.1±16.7 (22–73)	
Median	48.5	50.5	36.5	
Male gender, n (%)	21 (33.9)	19 (36.5)	2 (20)	0.47
Female gender, n (%)	41(67.1)	33 (63.5)	8 (80)	
Single nodule, n (%)	17 (27.4)	13 (25)	4 (40)	0.44
Goiter, n (%)	45 (72.6)	39 (75)	6 (60)	
Nodule location				0.47
Left, n (%)	36 (42.8)	33 (45.2)	3 (27.3)	
Right, n (%)	44 (52.4)	37 (50.7)	7 (63.6)	
Isthmus, n (%)	4 (4.8)	3 (4.1)	1 (9.1)	
Nodule size (mm)				0.24
Mean +/- SD	20.1±8.9	20.5±8.8	17.2±9.5	
Range	6–50	6.9–50	6–35	
Median	18	18.4	17	
*Scintigraphy of nodule				0.24
Hypofunctioning, n (%)	36 (64.3)	29 (60.4)	7 (87.5)	
Indifferent, n (%)	20 (35.7)	19 (39.6)	1 (12.5)	
Cytology of nodule, n (%)	71 (84.5)	66 (91.7)	6 (8.3)	
Histology of nodule, n (%)	32 (38.1)	21 (65.6)	11 (34.4)	
FNA+Operation, n (%)	19 (22.6)	14 (70)	6 (30)	
Strain elastography Score Transverse				0.0076
ES 1, n (%)	0 (0)	0 (0)	0 (0)	
ES 2, n (%)	54 (64.3)	51 (69.9)	3 (27.3)	
ES 3, n (%)	26 (30.9)	20 (27.4)	6 (54.5)	
ES 4, n (%)	4 (4.8)	2 (2.7)	2 (18.2)	
Strain elastography Score Longitudinal				0.0084
ES 1, n (%)	0 (0)	0 (0)	0 (0)	
ES 2, n (%)	53 (63.1)	49 (67.1)	4 (36.4)	
ES 3, n (%)	28 (33.3)	23 (31.5)	5 (45.4)	
ES 4, n (%)	3 (3.6)	1 (1.4)	2 (18.2)	
Strain value				0.76
Mean +/- SD	0.24±0.12	0.24±0.12	0.23±0.13	
Range	0.06–0.67	0.06–0.67	0.07–0.48	
Median	0.22	0.22	0.22	
Strain ratio				0.83
Mean +/- SD	1.73±1.19	1.74±1.23	1.68±0.9	
Range	0.37–7.88	0.37–7.88	0.52–2.86	
Median	1.41	1.38	1.82	
ARFI-Imaging				0.0034
Mean +/- SD	1.75±0.59	1.64±0.47	2.55±0.73	
Range	0.67–3.77	0.67–2.97	1.4–3.77	
Median	1.66	1.6	2.45	
2D-SWE				0.0075
Mean +/- SD	2.53±0.59	2.47±0.54	2.97±0.72	
Range	1.4–4.1	1.4–4.1	1.8–4	
Median	2.5	2.4	2.9	

### Cytology/Histology

**Fig 4:**
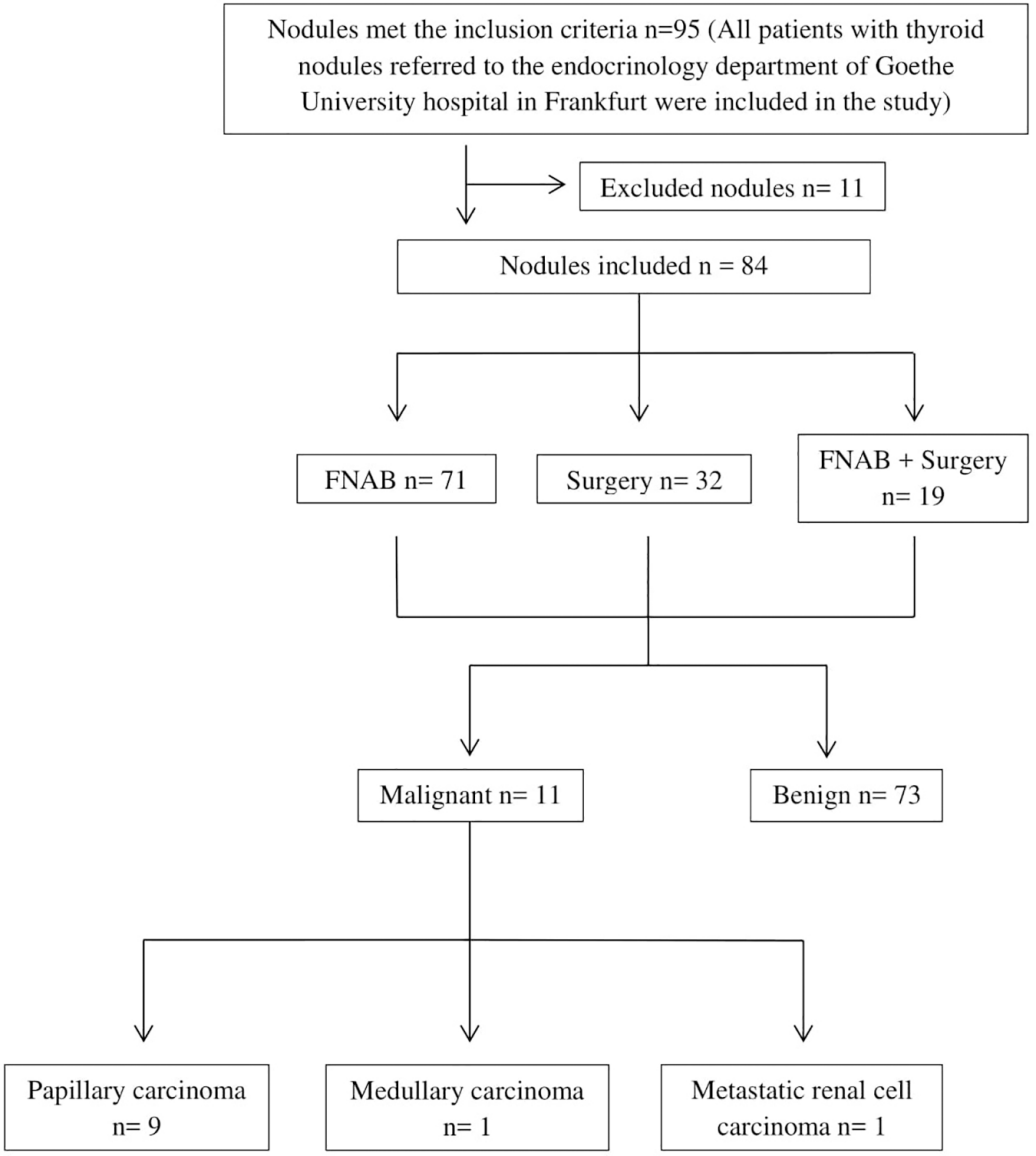
Number of nodules that underwent FNAB and/or surgery, number of benign and malignant thyroid nodules and types of thyroid cancer.

FNAB was performed on 71 nodules. 9 of these nodules were referred for surgery because of malignant or indeterminate cytology. 23 additional nodules were referred for surgery. Surgery was advised for goiters with multiple nodules or nodules with suspicious sonographic/elastographic features and benign FNAB. Overall, 32 nodules were treated surgically. Histology revealed benign adenomas and/or regressive changes in 21 of these nodules, papillary carcinoma in 9 nodules, medullary thyroid carcinoma in 1 nodule, and metastatic renal cell carcinoma 1 nodule (Fig 4).

### B-Mode ultrasound

Thyroid nodules were evaluated for echogenicity, echotexture, the presence/absence of a halo sign, and the presence/absence of microcalcification and/or macrocalcification. The results of the B-Mode ultrasound are displayed in detail in Table 2.

**Table 2:** Sensitivity, specificity, PPV and NPV for malignant thyroid nodules for different ultrasound patterns (including SE, ARFI and 2D-SWE).

B-mode	Criteria	Benign (n = 73)	Cancer (n = 11)	Sens (%)	Spec (%)	NPV (%)	PPV (%)	+LR	-LR
	Hypoechogeniticity								
	Yes	36 (49.3)	8 (72.7)	72.7	50.7	92.5	18.2	1.5	0.5
	No	37 (50.7)	3 (27.3)	(39;94)	(38.7;62.6)	(79.6;98.4)	(8.2;32.7)	(0.9;2.3)	(0.2;1.4)
	Microcalcifications								
	Yes	25 (34.2)	8 (72.7)	72.7	65.7	94.1	24.2	2.1	0.4
	No	48 (65.8)	3 (27.3)	(39;94)	(53.7;76.5)	(83.7;98.8)	(11.1;42.2)	(1.3;3.4)	(0.1;1.1)
	Macrocalcifications								
	Yes	8 (11)	1 (9.1)	9.1	89	86.7	11.1	0.8	1
	No	65 (89)	10 (90.9)	(0.2;41.3)	(79.5;95.1)	(76.8;93.4)	(0.2;48.2)	(0.1;6)	(0.8;1.2)
	Absent Halo								
	Yes	65 (89)	11 (100)	100	10.96	100	14.47	1.1	0
	No	8 (11)	0 (0)	(71.5;100)	(4.9;20.5)	(63.1;100)	(7.5;24.4)	(1;1.2)	(0;0)
	Irregular margins								
	Yes	15 (20.5)	7 (63.6)	63.64	79.45	93.55	31.82	3	0.4
	No	58 (79.5)	4 (36.4)	(30.8;89.1)	(68.4;88)	(84.3;98.2)	(13.9;54.9)	(1.6;5.8)	(0.2;1)
	Oval shape								
	Yes	55 (75.3)	5 (45.5)	45.4	24.6	75	8.3	0.6	2.2
	No	18 (24.7)	6 (54.5)	(16.7;76.6)	(15.3;36.1)	(53.3;90.2)	(2.7;18.4)	(0.3;1.1)	(1.1;4.3)
	Pattern 3–4 Vask. *								
	Yes	12	5	50	82.4	91.8	29.4	2.8	0.6
	No	56	5	(18.7;81.3)	(71.2;90.5)	(81.9;97.3)	(10.3;56)	(1.3;6.3)	(0.3;1.1)
SE	ES 3–4 transverse								
	Yes	22	8	72.7	69.8	94.4	26.6	2.4	0.4
	No	51	3	(39;94)	(58;80)	(84.6;98.8)	(12.3;45.9)	(1.4;3.9)	(0.1;1)
	ES 3–4 longitudinal								
	Yes	24	7	63.6	67.1	92.4	22.6	1.9	0.5
	No	49	4	(30.8;89)	(55.1;77.6)	(81.8;97.9)	(9.6;41.1)	(1.1;3.3)	(0.2;1.2)
	Strain value nodule <0,135								
	Yes	14	4	36.4	80.8	89.4	22.2	1.9	0.8
	No	59	7	(10.9;69.2)	(69.9;89.1)	(79.4;95.6)	(6.4;47.6)	(0.8;4.7)	(0.5;1.2)
	Strain ratio >2.49								
	Yes	11	4	36.4	84.9	89.9	26.7	2.4	0.7
	No	62	7	(10.9;69.2)	(74.6;92.2)	(80.2;95.8)	(7.8;55.1)	(0.9;6.3)	(0.5;1.2)
ARFI**	Median-ARFI nodule >1,98 (m/s)								
	Yes	15	9	90	79.2	98.3	37.5	4.3	0.1
	No	57	1	(55.5;99.8)	(68;87.8)	(90.8;99.9)	(18.8;59.4)	(2.6;7.1)	(0;0.8)
	Median Ratio-ARFI <0,94								
	Yes	26	9	90	63.9	97.9	25.7	2.5	0.2
	No	46	1	(55.5;99.8)	(51.7;74.9)	(88.7;99.9)	(12.5;43.3)	(1.7;3.6)	(0.02;1)
2D-SWE	Median-MW-SWE nodule >2,65 (m/s) (21.07kPa)								
	Yes	24	8	72.7	67.1	94.2	25	2.2	0.4
	No	49	3	(39;94)	(55.1;77.7)	(84.1;98.8)	(11.5;43.4)	(1.4;3.6)	(0.2;1.1)
	Median-Ratio-MW-SWE <0,91								
	Yes	29	7	63.6	60.3	91.7	19.4	1.6	0.6
	No	44	4	(30.8;89.1)	(48.1;71.6)	(80;97.7)	(8.2;36)	(0.9;2.7)	(0.3;1.3)

SE = strain elastography, ARFI = Acoustic Radiation Force Impulse Imaging, 2D-SWE = 2D Shear Wave Elastography, ES = elastography score, Sens = sensitivity, Spec = Specificity, PPV = positive predictive value, NPV = negative predictive value, LR = likelihood ratio, vasc. = vascularisation

*Duplex ultrasound sonography was performed in 78 nodules.

**ARFI was performed in 82 nodules.

### Strain elastography (SE)

Strain elastography score ES-1 on transverse ultrasound imaging was found in no nodule; ES-2 in 54 nodules (51 benign nodules, 2 papillary carcinomas and one metastatic renal cell carcinoma); ES-3 in 26 nodules (20 benign nodules, 5 papillary carcinomas and one medullary carcinoma); and ES-4 in 4 nodules (2 benign nodules and 2 papillary carcinomas). Thus, 51 of 73 nodules (70%) with the final diagnosis of a benign nodule showed ES 1–2, and 8 of 11 nodules (73%) with the final diagnosis of thyroid cancer showed ES 3–4.

Sensitivity, specificity and NPV of SE using ES 3–4 for the diagnosis of malignant thyroid nodules and ES 1–2 for the diagnosis of benign thyroid nodules are shown on Table 2. No significant difference was found between the sensitivity and specificity of SE on transverse and longitudinal ultrasound imaging (p = 0.65). Details are shown in Table 2. AUROC (Area under the Receiver Operating Characteristic) for the diagnosis of malignant thyroid nodules using SE was 0.52 [95-CI: 0.32; 0.72] (p = 0.87). The optimal cut-off with the highest sum of sensitivity and specificity (Youden cut-off) for strain value was 0.135. AUROC for the ratio of SE in the nodule and healthy thyroid tissue for the diagnosis of malignant thyroid nodules was 0.52 [95-CI: 0.30; 0.74] (p = 0.84). The optimal cut-off (Youden cut-off) for strain ratio was 2.49 (Table 1). For SE, no significant difference was found between AUROC of the nodule and SE ratio (p = 0.91). Details are shown in Table 2.

### Acoustic Radiation Force Impulse (ARFI)-Imaging

In the present study a significant difference in median velocity of ARFI was found between healthy thyroid tissue {1.74 (1.33, 2.68)} and malignant thyroid nodules {2.44 (1.40, 3.77)} (p = 0.004), as well as between benign {1.60 (0.67, 2.97)} and malignant thyroid nodules (p<0.001), while no significant difference was found between healthy thyroid tissue {1.73 (1.11, 2.65)} and benign thyroid nodules (p = 0.108). Median velocity in healthy thyroid tissue was measured separately for patients with benign and malignant nodules. ARFI was performed on 82 nodules. AUROC for ARFI for the diagnosis of malignant thyroid nodules was 0.86 [95-CI: 0.72; 0.99] (p<0.001). The optimal cut-off with the highest sum of sensitivity and specificity (Youden cut-off) for ARFI measurements in thyroid nodules was 1.98 m/s. AUROC for the ratio of ARFI in the nodule and healthy thyroid tissue for the diagnosis of malignant thyroid nodules was 0.81 [95-CI: 0.69; 0.93] (p<0.001). The sensitivity, specificity and NPV for ARFI are shown in Table 2. The optimal cut-off (Youden cut-off) for ARFI ratio was 0.94 m/s. For ARFI, no significant difference was found between AUROC of the nodule and ARFI ratio (p = 0.33).

### 2D-Shear Wave elastography (2D-SWE)

A significant difference in median velocity of 2D-SWE was found between healthy thyroid tissue {0.41 (0.18, 1.10)} and benign thyroid nodules {2.40 (1.40, 4.10)} (p<0.001), as well as between healthy thyroid tissue {0.44 (0.22, 0.64)} and malignant thyroid nodules {3.00 (1.80, 4.00)} (p<0.001), and between benign and malignant thyroid nodules (p = 0.03). Median velocity in healthy thyroid tissue was measured separately for patients with benign and malignant nodules. AUROC for 2D-SWE of the thyroid nodule for the diagnosis of malignant thyroid nodules was 0.71 [95-CI: 0.49; 0.92] (p = 0.051). The optimal cut-off value for velocity with the highest sum of sensitivity and specificity (Youden cut-off) for SWE measurements in thyroid nodules was 2.65 m/s, and 21.07 kPa for elasticity (Table 2). The sensitivity, specificity and NPV for 2D-SWE are shown in Table 2. AUROC for the ratio of 2D-SWE in the nodule and healthy thyroid tissue for the diagnosis of malignant thyroid nodules was 0.66 [95-CI: 0.47; 0.85] (p = 0.096). The optimal cut-off (Youden cut-off) for 2D-SWE ratio was 0.91m/s (Table 2). For 2D-SWE, no significant difference was found between AUROC of the nodule and 2D-SWE ratio (p = 0.50).

### Comparison of SE, ARFI-Imaging and 2D-SWE

**Fig 5:**
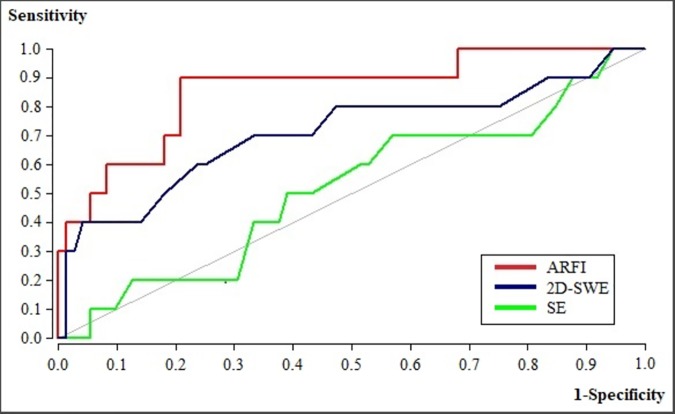
Comparison of ROC-Curves for SE, ARFI and 2D-SWE.

A significant difference in AUROC was found between SE, ARFI and 2D-SWE (0.52 vs. 0.86 vs. 0.71, p = 0.029), which was due to the low AUROC of SE (Fig 5). No significant difference in AUROC was found between ARFI and 2D-SWE (0.86 vs. 0.71, p = 0.31), or 2D-SWE and SE (0.71 vs. 0.52, p = 0.26). A significant difference in AUROC was found between ARFI and SE (0.86 vs. 0.51, p = 0.008). No significant difference in AUROC was found between the SE, ARFI and 2D-SWE ratios (0.52 vs. 0.81 vs. 0.66, p = 0.14). Sensitivity, specificity and NPV using 0.135, 1.98 m/s and 2.65 m/s as cut-off values for the diagnosis of malignant thyroid nodules for SE, ARFI and 2D-SWE measurements respectively are shown in Table 2. While no significant difference was found between the specificities of SE, ARFI and 2D-SWE (p = 0.11), a significant difference was found between the sensitivities of SE, ARFI and 2D-SWE (p = 0.029), which was due to the low sensitivity of SE.

## Discussion

The strength of the present study is the comparison of three different ultrasound-based techniques, namely (1) strain elastography, (2) point shear wave elastography using ARFI and (3) 2D-shear wave elastography, for the differentiation of thyroid nodules in the same patient population, using cytological/histological assessment as a reference method. To our knowledge, only one recently published study has compared all three elastography methods and showed that ARFI and 2D-SWE had better diagnostic performance compared with SE and conventional ultrasound [18]. Previous thyroid gland elastography studies evaluated either SE as a qualitative elastography method, or ARFI and 2D-SWE as quantitative elastography methods for the differentiation between benign and malignant thyroid nodules.

The results of the present study show comparable results for ARFI and 2D-SWE for the differentiation of benign and malignant thyroid nodules, with no significant difference found in AUROC between ARFI and 2D-SWE (0.86 vs. 0.71, p = 0.31). Moreover, 2D-SWE showed results comparable with SE, with no significant difference in AUROC between 2D-SWE and SE (0.52 vs. 0.71, p = 0.26). The results for SE were not comparable and less reliable, with a significantly lower AUROC compared with ARFI (0.52 vs. 0.86, p = 0.008). While no significant difference between SE, ARFI and 2D-SWE (p = 0.11) was found for specificity to diagnosis of malignant nodules, a significant difference was found for sensitivity (p = 0.029), which was due to the significantly lower sensitivity of SE. The results and these discrepancies might be explained by the limited sample size of nodules and especially malignant nodules and by the operator dependency of elastography. Lam et al. reported a significant influence of precompression on the measurements using 2D-SWE in normal thyroid tissue, benign nodules and papillary carcinomas [19], which could explain the variation and discrepancies in published data of several studies of 2D-SWE in thyroid nodules. Elastography is an operator-dependent technique, therefore measurement bias resulting from clinicians using different examination techniques may exist. The results of elastography can be affected by several factors such as the experience of the examiner, nodule characteristics (rather large or small size, isthmic or paratracheal position, cystic components and classifications) and artifacts such as the carotid artery pulsation. Follicular carcinomas may be soft in elastography, which can lead to false negative results [4]. FNAB is then useful in nodules with indeterminate elastography and can increase the detection rate of malignancy. On the other hand, elastography is a valuable tool in the management of patients with indeterminate or non-diagnostic cytology. The results of our study indicate that ARFI and 2D-SWE are comparable in the evaluation of thyroid nodules. ARFI is superior and more reliable compared with SE. Unlike sensitivity or specificity, PPV depends on the characteristics of the population under study and the prevalence of the examined disease. PPV decreases as the prevalence of the disease decreases. The very low prevalence of thyroid cancer explains the low PPV of the three elastography methods in our study [20].

Many patients with thyroid nodules receive unnecessary FNAB or surgery. Moreover, there is variation in the reported sensitivity and specificity of FNA which could miss up to a third of all thyroid malignancies [21]. Elastography could therefore be integrated in the diagnostic algorithm of thyroid nodules and can be useful to select suspicious nodules for FNAB or surgery. Our study showed that ARFI and 2D-SWE might be helpful and have a significant contribution in the differentiation between benign and malignant nodules, and could have an application prospect in clinical practice.

As reported by recent WFUMB guidelines, TIRADS may be useful in the stratification of malignancy risk of thyroid nodules in clinical practice [22]. The classification system TIRADS was first used by Horvath et al. [23]. However, the described ultrasound patterns were difficult to apply in all thyroid nodules. The TIRADS by Kwak et al. is simple to apply and enables stratification of the malignancy risk of thyroid nodules [24]. TIRADS categories 4a, 4b, 4c and 5 refer to nodules with one or more suspicious features. Nodules classified as TIRADS 4 or 5 showed higher probability of malignancy [25] [26]. In our study, the TIRADS classification and features were not applied, since this was outside the aims of the study. Nevertheless, TIRADS can be a useful tool in the diagnostic algorithm of thyroid nodules, and should be further investigated in future studies.

### Results of SE in comparison to previous studies

A meta-analysis reported a sensitivity of 92% and a specificity of 90% for SE for the diagnosis of malignant thyroid nodules [4]. The sensitivity, specificity and NPV for strain ratio of SE in the present study using a cut-off value of > 2.49 were 36.4%, 84.9% and 89.9% respectively, and were lower than in the study of Cantisani et al., which reported a sensitivity, specificity and NPV of 97.3%, 91.7% and 98.2% respectively, for the prediction of malignancy using a strain ratio ≥2[27]. Another study with 158 nodules in 138 patients reported a NPV of 95% for SE using ES 3&4 for the diagnosis of malignant thyroid nodules, and ES 1&2 for the diagnosis of benign thyroid nodules, which was comparable to the NPV of 92.4% in the present study. Sensitivity and specificity of SE in the present study was lower than in the above-mentioned study, with a sensitivity of 72.7% vs. 76% and a specificity of 69.8% vs. 72%, respectively [28]. Vorlaender et al. reported a NPV of 100% when using a cut-off strain value above 0.31 for SE [29]. Another study showed that the specificity and AUROC of the semi-quantitative strain ratio evaluation was higher than the qualitative elastography score (0.88 vs. 0.79, p <0.001) [30]. AUROC of SE in the present study was lower than in the previously mentioned study, with an AUROC value of 0.52 vs. 0.79 [30]. The results of SE in the present study were higher than in the study of Moon et al., with a sensitivity of 72.7% vs. 65% and a specificity of 69.8% vs. 58% [31]. Cantisani et al. reported a sensitivity of 90.6%, specificity of 93%, PPV of 82.8%, NPV of 96.4% and accuracy of 92.4% using the strain ratio evaluation. In their study, strain ratio helped identify 82.5% malignant nodules that were considered to be benign before the application of strain ratio [32]. The results of the meta-analysis of Razavi et al. showed a better diagnostic performance for strain ratio compared with elasticity score in the characterization of thyroid nodules [33]. The sensitivity of SE in the present study was lower than in the meta-analysis of Hu et al., with a sensitivity of 72.7% vs. 84%. In this meta-analysis, the sensitivity of SE was higher than that of SWE. The low sensitivity of SE in our study compared with other studies, and the fact that the results for SE were not comparable and less reliable, with a significantly lower AUROC compared to ARFI, might be explained by the limited sample size of nodules and especially malignant nodules in our study, by the possible selection bias in our study, and by the operator dependency of elastography. Elastography is an operator-dependent technique, therefore measurement bias resulting from clinicians using different examination techniques may exist. Nevertheless, SE is a reliable elastography method that showed promising results in the above-mentioned studies. However, in the present study the results of ARFI were superior to SE [34].

### Results of pSWE using ARFI in comparison to previous studies

Sensitivity and specificity of ARFI in the present study were lower than in a recent study, with a sensitivity of 90% vs. 96.8% and a specificity of 79.2% vs. 95.7% [35]. Sensitivity of ARFI in the differentiation between benign and malignant thyroid nodules in the present study is similar to the 75–100% reported in previous studies, and specificity is lower than the 82.2–96.2% reported in other studies [35][36][37][38]. A previous study with 60 nodules in 55 patients showed that the combination of SE with ARFI-Imaging improved specificity for the diagnosis of malignant nodules from 72% to 92% but reduced sensitivity from 76% to 58% [7]. The best cut-off point was 1.98 m/s in the present study, lower than the 2.55–2.84 m/s reported in previous studies [27][35][36]. This shows that a lower velocity value does not necessarily indicate a benign nodule, and is therefore relevant in clinical practice. Liu et al. reported a cut-off value of 2.15 m/s for malignant thyroid nodules without highly suspicious features on ultrasound. Zhang et al. reported a velocity >3.10 m/s to be an independent risk factor in predicting papillary thyroid microcarcinoma[39]. AUROC for ARFI for the diagnosis of malignant thyroid nodules was 0.86 in the present study, similar to the 0.861 reported in the study of Zhang et al. [38], and higher than in two other studies, with AUROC values of 0.86 vs. 0.828 and 0.86 vs. 0.69, respectively [26][40]. AUROC in the present study was lower than the 0.964–0.989 reported in other studies [35][37].

### Results of 2D-SWE in comparison to previous studies

The results of our study showed that the optimal cut-off value for velocity for 2D-SWE for predicting malignancy was 2.65 m/s, with a sensitivity and specificity of 72.7% and 67.1% respectively, and a NPV of 94.2%. The optimal cut-off value for elasticity was 21.07 kPa. The NPV in the present study was higher than in a previous study with 64 nodules (94.2% vs. 86.7%). Sensitivity was higher and specificity lower compared with the study in question (72.7% vs. 68.4% and 67.1% vs. 86.7%, respectively) [41]. Sensitivity of 2D-SWE in the present study was comparable to the sensitivity determined in previous studies, while specificity and cut-off values were lower. In previous studies, sensitivity and specificity were 66.6–85.2% and 71.1–93.9% respectively, and cut-off values ranged from 34.5 to 66kPa in the differentiation between benign and malignant thyroid nodules [8][41][42][43][44]. AUROC of 2D-SWE in the present study was 0.71, and was lower than the 0.84–0.829 reported in other studies [40][41]. The results and these discrepancies might be explained by the limited experience of the examiners with 2D-SWE.

The present study has some limitations: The malignant nodules were predominantly papillary carcinomas, which might limit the diagnostic utility to this entity. The study had a limited sample size of nodules, especially malignant thyroid nodules, and no follicular carcinomas were included. The limited sample size of nodules might not allow the calculation of reliable cut-off values for the three elastography methods. The fact that follicular carcinoma accounts only for 15% of thyroid cancer makes papillary carcinoma more likely to be detected and included in a study, investigating thyroid nodules, especially when the sample size of the nodules is limited as in our study. Selection bias may exist, since patients included in our study were scheduled for FNAB for thyroid nodules with suspicious ultrasound features, or the largest one of multiple thyroid nodules that did not necessarily have any suspicious ultrasound features. Patients with suspicious or malignant cytology were referred to surgery, some patients though refused FNAB and received thyroid surgery as a first-line treatment.

In the literature there are variable proposed cut-off values for the differentiation between benign and malignant thyroid nodules with the three elastography methods. These discrepancies are explained by the fact that there are several criteria for the selection of the most appropriate cut-off value in a diagnostic test. The calculation of a cut-off value depends on the prevalence of the malignant thyroid nodules, the population of the study and the sample size of nodules. In some cases, sensitivity is more important than specificity, in other cases, specificity may be preferred over sensitivity. The selection of the most appropriate cut-off value is based on the situation the test is applied to and the importance of the test sensitivity compared to specificity. In our study we selected the cut-off values with the highest sum of sensitivity and specificity for the three elastography methods. Therefore, the cut-off values of the elastography methods should be interpreted and used in clinical practice only in combination with the results of the B-mode ultrasound examination.

In conclusion, our study is among the first to compare all three elastography methods, namely SE, pSWE using ARFI and 2D-SWE, in the same study population. Our study demonstrates comparable results for ARFI and 2D-SWEfor the differentiation of thyroid nodules. SE showed a low sensitivity and specificity and the results were not comparable to ARFI. Our study indicates that ARFI and 2D-SWE are comparable in the evaluation of thyroid nodules. ARFI is superior and more reliable compared with SE. These discrepancies might be explained by the limited sample size of nodules and especially malignant nodules, and by the operator dependency of elastography. Further studies with more nodules and varied tumor types are required to validate the study results.
